# Exploring Doping Awareness: Medical Experts’ Perspectives and Their Commitment to Doping Prevention

**DOI:** 10.3390/pharmacy13030059

**Published:** 2025-04-24

**Authors:** Vanya Rangelov Kozhuharov, Radiana Staynova, Kalin Ivanov, Hristo Manev, Stanislava Ivanova

**Affiliations:** 1 Department of Pharmacognosy and Pharmaceutical Chemistry, Faculty of Pharmacy, Medical University of Plovdiv, 4002 Plovdiv, Bulgaria; vanya.kozhuharov@mu-plovdiv.bg (V.R.K.); kalin.ivanov@mu-plovdiv.bg (K.I.); 2 Research Institute, Medical University of Plovdiv, 4002 Plovdiv, Bulgaria; 3 Department of Organisation and Economics of Pharmacy, Faculty of Pharmacy, Medical University of Plovdiv, 4002 Plovdiv, Bulgaria; radiana.staynova@mu-plovdiv.bg; 4 Department of Medical Physics and Biophysics, Faculty of Pharmacy, Medical University of Plovdiv, 4002 Plovdiv, Bulgaria; hristo.manev@mu-plovdiv.bg

**Keywords:** doping, WADA, doping prevention, professional sport, prohibited list, pharmacists, medical doctors, unintentional doping, health

## Abstract

Humanity has used and explored various performance-enhancing remedies since ancient times. To protect clean sport, athletes’ health, and to provide fair and transparent competitions, different anti-doping policies were implemented. Nowadays, the anti-doping policies are evolving every year and are governed by the World Anti-Doping Agency. The use of doping in sports is regarded as a persistent problem across various athletic disciplines; at the same time, the topic of whether doping is preventable is highly discussed. The aim of this study was to assess the knowledge of medical specialists about doping compounds and to analyze their willingness and preparedness to participate in doping prevention programs. A cross-sectional questionnaire-based study was conducted between March 2024 and May 2024. Study participants included medical and pharmacy students, pharmacists, and medical doctors. Statistical analysis was performed using IBM SPSS Statistics version 24.0. Descriptive statistics, one-way analysis of variance (ANOVA), and independent-samples t-test were applied to analyze different variables. The results from the study suggested that healthcare professionals, particularly pharmacists, could be successfully engaged in doping prevention. Additionally, community pharmacies could also be involved in doping-prevention strategies since they are recognized as easily accessible healthcare locations. The relationship between medical specialists and professional athletes is essential for raising awareness, receiving trustworthy information, and developing decision-making capability to prevent not only the intentional but also the unintentional anti-doping rule violations.

## 1. Introduction

Humanity has used and explored a great variety of performance-enhancing remedies since ancient times. Some of these remedies included Peyote juice [1], Catha edulis leaves [2], dried figs, mushrooms, etc. However, the word “doping” is not so old. It was mentioned for the first time only in 1889 in an English dictionary [3]. Originally, this word described a mixture containing opium, which was used to “dope” horses. At the beginning of the 20th century, “doping” was regularly used to describe the illegal drugging of racehorses [3].

With the evolution of the modern pharmaceutical industry in the 20th century, many athletes began to experiment with different cocktails of pharmacologically active compounds to improve strength and speed, to overcome fatigue, and to achieve victory. Although this practice was dangerous, it was not illegal until the mid-20th century. Many cases of fatal incidents with professional athletes involving the intake of doping were reported in this period [4]. To protect clean sport, athletes’ health, and to provide fair and transparent competitions, some anti-doping policies were implemented gradually. The obligatory doping testing was introduced for the first time by the International Olympic Games Committee in 1966 [5,6]. Nowadays, the governance of the anti-doping policies is evolving every year and belongs to the World Anti-Doping Agency (WADA)—an independent international agency composed of and funded equally by the Olympic Movement and Governments of the world. WADA was established in 1999 and has the important mission to lead a collaborative worldwide movement for doping-free sport across all sports and countries [7]. WADA provides some very important documents for professional sport, including “A Code of Ethics” and “The List of Prohibited Substances and Methods (List)”. The list describes what compounds and methods are prohibited in sport and when. It is updated annually [8]. Although many substances regarded as doping can significantly improve athletic performance, their intake is prohibited for several important reasons: intake could be harmful to the athletes, and the use of doping compounds makes the competition inequitable.

Nowadays, the use of performance-enhancing drugs in sports is regarded as a persistent problem across various athletic disciplines [9]. Athletes often resort to doping in an attempt to gain a competitive edge, leading to unfair advantages and compromising the principles of fair play [10,11]. However, many cases of unintentional doping were also reported recently [12].

The topic of whether doping is preventable is highly discussed. Many different strategies for doping prevention have been developed in the past years, and it is expected that some novel approaches for doping prevention will be introduced in the next few years. Essential key points in the prevention of intentional or inadvertent doping are the constant education of athletes and the possibility to receive support from medical specialists or other athletes. In general, doping prevention strategies are complex and are based on a multidisciplinary approach.

Currently, some apps have been introduced that could support doping prevention:

- FitCoach Version 10.3.0: Designed specifically for athletes. FitCoach offers personalized training plans, nutritional guidance, and performance tracking tools to help athletes reach their goals without resorting to doping [13].
- Clean Sport Version 2.1.1: This app focuses on education and prevention, offering athletes essential resources to stay informed about anti-doping regulations, with features such as medication tracking, personalized content, and urgent updates [14].
- Sport Integrity Version 5.0.6: This app aims to promote clean and fair play in sports by providing athletes with information on anti-doping policies, educational resources, and reporting mechanisms for suspected doping activities [15].

Important roles in doping prevention strategies could be played by different medical specialists, including medical doctors, pharmacists, and others. However, the medical specialists who could be involved in these programs must have additional knowledge about the prohibited substances and methods. This knowledge will enable them to play a crucial role in educating athletes about the risks and consequences of doping, as well as assisting in doping control programs. By understanding the specific substances and methods that are prohibited, medical specialists can effectively guide athletes in making informed decisions about their medication and dietary supplement (DS) use, ensuring compliance.

The aim of this study was to assess the knowledge of medical specialists (pharmacists, physicians, and students) about doping substances and to analyze their willingness to participate in doping prevention programs. This is the first study performed in Bulgaria that evaluates the preparedness and willingness of medical specialists to participate in doping prevention programs.

## 2. Materials and Methods

### 2.1. Study Design, Participants, and Setting

A cross-sectional questionnaire-based study was conducted between March 2024 and May 2024. Study participants included medical and pharmacy students (3rd to 6th year) from the Faculty of Medicine and Faculty of Pharmacy, Medical University of Plovdiv; pharmacists who were members of Regional Pharmaceutical Chamber Plovdiv of the Bulgarian Pharmaceutical Union (BPhU); and medical doctors who worked at Medical University of Plovdiv and University hospitals in the city of Plovdiv, Bulgaria. Plovdiv is the second biggest city in Bulgaria.

### 2.2. Study Tool

A questionnaire was developed by the investigators who are experts in pharmaceutical chemistry, pharmaceutical analysis, pharmacy practice, and pharmaceutical legislation. An initial draft of the questionnaire was evaluated by two researchers to ensure accuracy, structure, content clarity, and correct grammar. Then, a pilot study involving 10 participants assessed the instrument’s face and content validity. Readability and completion time were also evaluated. Adjustments were incorporated in the revised version of the questionnaire according to participants’ feedback. The pre-tested samples were excluded from the final analysis.

The final version of the study tool contained 34 questions divided into three sections. The questionnaire is presented in the Supplementary Data (Supplementary File S1). The first section included questions regarding the socio-demographic characteristics and sporting habits of study participants. The second section referred to the knowledge and awareness of respondents about prohibited substances and methods in sports. The last section addressed a willingness to participate in different anti-doping activities and campaigns. A Google Forms platform was used for the distribution of the survey. The survey tool consisted of multiple-choice, closed-ended questions, and some open-ended questions requiring a short answer.

The assessment of anti-doping knowledge is determined by calculating a cumulative score, obtained by summing the points assigned for each correct answer. Two points were given to each completely correct answer; a partially correct answer received 1 point, while an incorrect answer was assigned 0 points. “Don’t know” options were considered incorrect answers. The total knowledge score was 36.

### 2.3. Data Collection

During the study period, there were 967 pharmacists who were members of the Regional Pharmaceutical Chamber Plovdiv of BPhU. An invitation including a statement with the aim of the study and a web link to the Google Forms platform was sent by e-mail to a random sample of 500 pharmacists. The same form of invitation was sent to the medical doctors at the university and the university hospitals. To increase the response rate, two email reminders were sent during the study period of three months.

Every student in the Medical University of Plovdiv has an account in MS Teams. The link to the web-based survey was sent via MS Teams. The questionnaire was distributed only to students from the 3rd to 6th year of their education since subjects like pharmacology and pharmaceutical chemistry begin to be studied in the third year.

At the beginning of the questionnaire, a consent statement was supplied, which included the objective of the study, an estimate of how long it would take, an explanation of the confidentiality of replies, and the voluntary nature of participation. Respondents gave their agreement by agreeing to start answering the questions in the online tool. Individual identifiers were not collected. The study on human participants was in accordance with national legislation and institutional requirements. Approval from an ethics committee was deemed unnecessary for the conduct of this study. From a methodological standpoint, the survey was categorized as sociological, lacking any elements of clinical research. No personal data were retained or subjected to analysis.

### 2.4. Data Analysis

Statistical analysis was performed using IBM SPSS Statistics version 24.0 (IBM Corp, Armonk, NY, USA). Descriptive statistics, one-way analysis of variance (ANOVA), and independent-samples *t*-test were applied to analyze different variables. For comparison of three or more groups, Tukey’s post hoc test was used. A *p*-value less than 0.050 was considered to be statistically significant.

## 3. Results

### 3.1. Socio-Demographic Characteristics and Sporting Habits

The present survey involved 585 respondents. More than half of them were female (63.4%); males accounted for 35.5%, and eight respondents (1.4%) chose not to answer this question. The mean age of respondents was 27.9 ± 8.9 years (range 20–76 years). The majority of respondents belong to the 20–29 age group, likely due to the predominance of students among the participants. The highest percentage was pharmacy students (32.8%), followed by pharmacists (24.1%). Physicians and medical students accounted for 43.1% of the respondents. With regard to sports participation, 241 respondents indicated that they are currently engaged in sports activities. A comparable percentage of individuals reported previous participation in sports (*n* = 261), while 14.2% of respondents indicated that they had never engaged in any sports activities. Over half of the respondents (66.8%) reported participating in amateur sports, whereas only 5.3% identified as professional athletes. One hundred and ninety-nine respondents had attended a lecture on doping, and most of these respondents were pharmacy students. Table 1 presents the demographic and main characteristics of the respondents.

### 3.2. Knowledge and Awareness About Prohibited Substances and Methods in Sports

**Figure 1:**
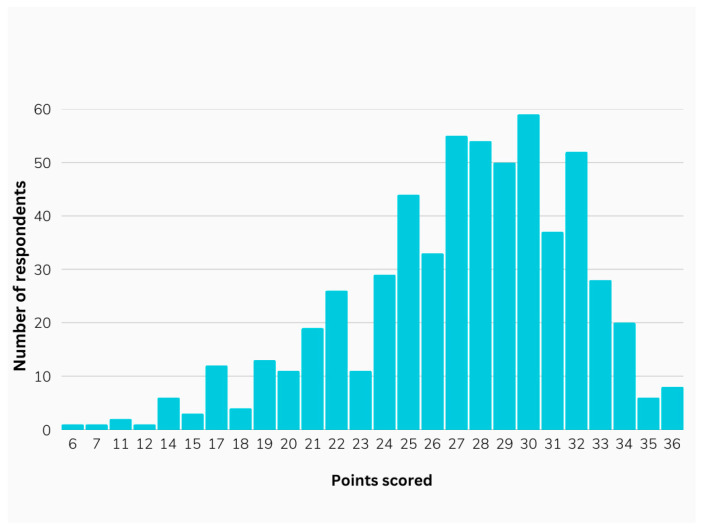
Distribution of participants’ scores.

The mean score for respondents’ knowledge regarding prohibited substances and methods in sport was 27.23 ± 4.82 (with a maximum score of 36), with scores ranging from 6 to 36 points. Figure 1 illustrates the distribution of scores in relation to the number of correct answers.

**Figure 2:**
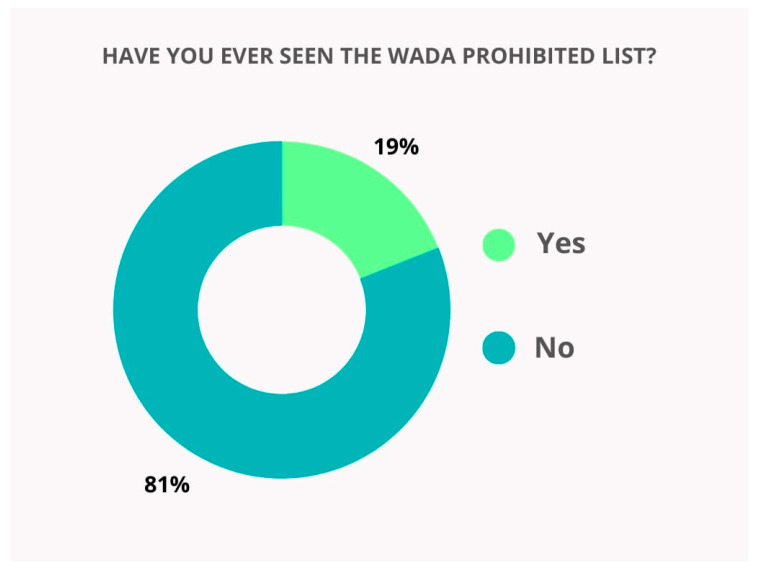
Distribution of respondents’ answers to the question “Have you ever seen WADA’s Prohibited List?”.

The majority of respondents (98.1%) were familiar with the term “doping”. A significant proportion of respondents (91.1%) gave a correct answer to the question of what WADA is. The remaining 8.8% either provided an incorrect answer to this question or indicated that they did not know. Most respondents reported being unfamiliar with the WADA Prohibited List, with only 19% having reviewed it (Figure 2).

**Figure 3:**
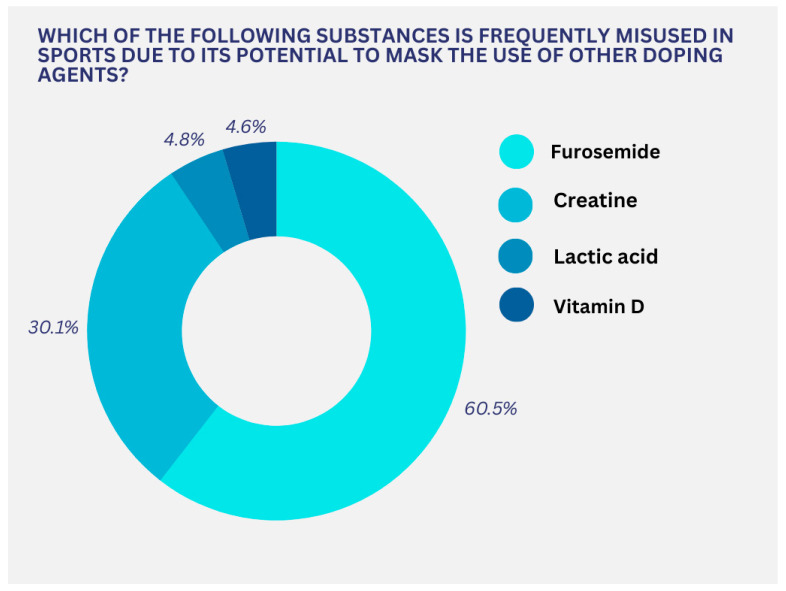
Respondents’ knowledge regarding masking agents used to conceal doping.

Four hundred and thirty respondents (73.5%) recognized anabolic steroids as doping substances, while 26.5% provided an incorrect answer. More than half of the respondents were aware that diuretics are prohibited due to their potential to mask the use of other doping substances, as 60.5% indicated furosemide as a correct answer (Figure 3).

**Figure 4:**
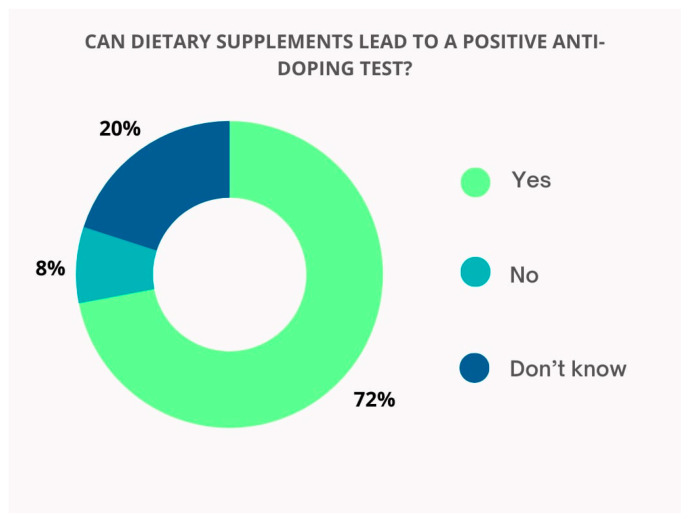
Respondents’ awareness of doping risks associated with DS use.

The majority of healthcare professionals/students are aware of the potential risks of taking DSs (72%) (Figure 4).

**Figure 5:**
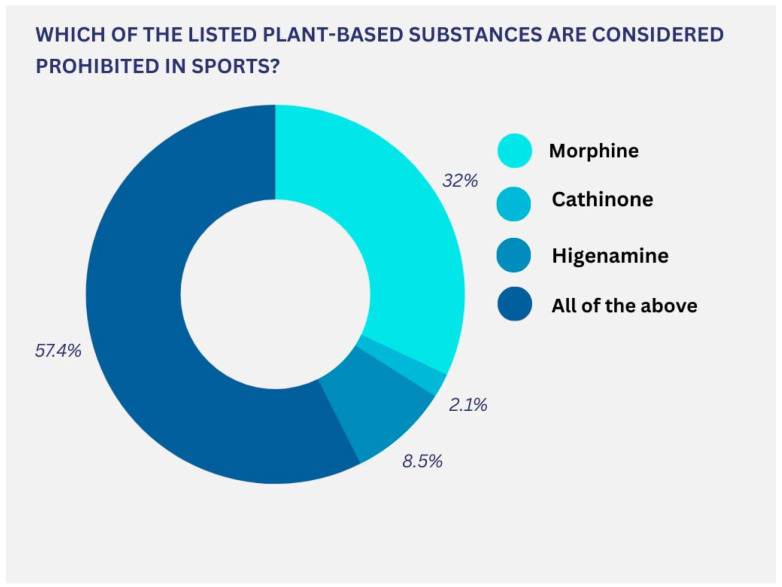
Respondents’ knowledge of prohibited plant-based substances in sports.

The use of plant-based DSs is gaining popularity due to their potential health benefits. Over 57% of medical professionals surveyed recognize substances prohibited in sport that are of plant origin (Figure 5).

To the question, “How can doping harm an athlete’s health?”, 418 respondents (71.5%) provided the correct answer. When asked which of the following substances are considered doping, 477 participants answered correctly. Of these, 62% indicated “corticosteroids”, 21% indicated meldonium, and 25% indicated human growth hormone. Additionally, 86.2% of the respondents recognized erythropoietin as a substance that could enhance athletic performance and is included on the WADA Prohibited List. A total of 88.72% mentioned ephedrine as commonly found in DSs, while only 25.3% correctly identified which plant could be a source of doping.

**Figure 6:**
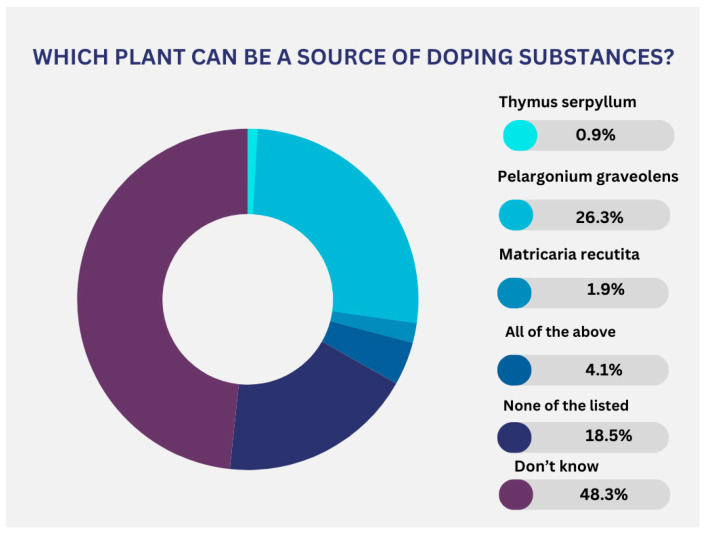
Respondents’ awareness of plant-based sources of doping substances.

Only 26.3% of respondents are aware that the intake of pelargonium graveolens may result in a positive test (Figure 6).

The independent samples *t*-tests for the two groups performed showed significant differences between the two groups studied, suggesting that whether an individual is a medical doctor or a pharmacist influences the number of correct answers concerning the doping topic (*p* < 0.009). A similar correlation was observed between the groups of medical students and pharmacy students, supporting our hypothesis that different professional orientations impact the results (Table 2).

Moreover, a one-way analysis of variance with Tukey’s post hoc for comparisons of the four groups with respect to the overall knowledge result was performed. The ANOVA results (F (3, 581) = [12.062], *p* < 0.001 *) show that there is a statistically significant difference between the result scores in the different groups. Using Tukey’s post hoc test, we confirmed the results obtained above that the mean value of questionnaire-based scores was significantly different between pharmacists and medical doctors (*p* = 0.028 *, 95% C.I. = [0.12, 3.11]) and between pharmacy students and medical students (*p* < 0.001 *, 95% C.I. = [0.99, 3.73]), respectively.

Respondents who reviewed WADA’s Prohibited List demonstrated higher knowledge than those who did not (*p* < 0.001). The results are presented in Table 3.

The majority of respondents (n = 502) have participated in or are currently engaged in sports, which influences their answers. The differences found between the groups are presented in Table 4.

### 3.3. Willingness to Participate in Different Anti-Doping Activities and Campaigns

**Figure 7:**
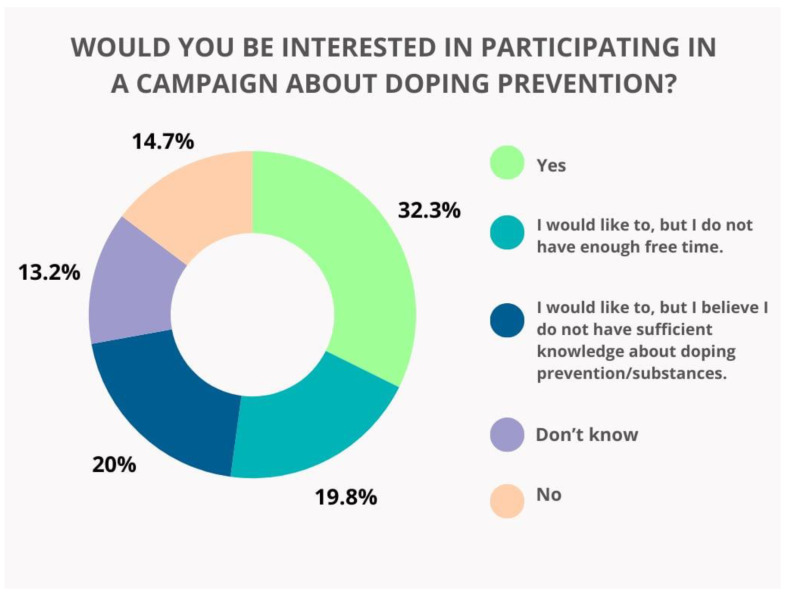
Respondents’ willingness to participate in campaigns about doping prevention.

Campaigns focused on doping prevention are of interest to 32.3% of the respondents, while 19.8% express interest but report a lack of enough free time. Additionally, 20% believe they do not have sufficient knowledge, and only 14.7% are not interested in such activities (Figure 7).

Only 8.2% of respondents reported having consulted an athlete regarding substances that enhance athletic endurance, while 10.1% had provided consultations about the side effects of such substances (Table 5). Additionally, 39.3% were aware of cases of unintentional doping, whereas 71.8% had no such information.

The *p*-values in Table 5 are part of the ANOVA results for the comparison of the pairs of indicators from the first column of the table, and they show that we observe statistically significant differences between the result scores in the respective pairs.

**Figure 8:**
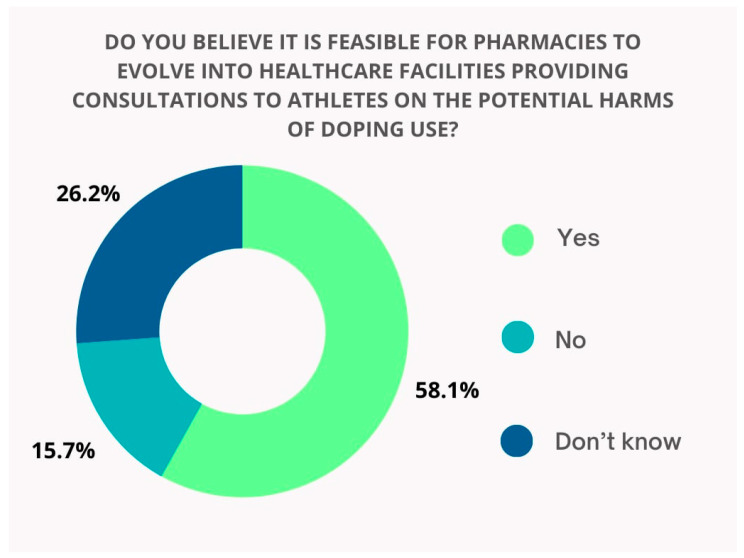
Frequency distribution of respondents based on their answers to the question.

More than half of the respondents were of the opinion that pharmacies could be turned into healthcare facilities to provide advice to athletes on the potential harms of doping (Figure 8).

## 4. Discussion

Medical specialists might play a crucial role in doping prevention strategies. The relationship between medical specialists and professional athletes is essential for raising awareness, receiving trustworthy information, and developing decision-making capability to prevent not only intentional but also unintentional anti-doping rule violations. However, the health specialists involved in doping-prevention projects need constant and specific training about prohibited substances and doping prevention. According to our study, most of the medical specialists are aware of doping substances and possess the expertise to prevent doping. Additionally, we found that 41.84% of pharmacists were consulted or provided education regarding doping substances, while the percentage of physicians was significantly less (13.9%). This may further indicate an influence on the number of correct answers provided. Similar studies conducted in Taiwan and Finland evaluated pharmacists’ anti-doping knowledge [16,17]. The Taiwanese study revealed that only 15.3% of pharmacists surveyed demonstrated the knowledge required to counsel professional athletes. In contrast, in Finland, this percentage was significantly higher, reaching 67.9%. Meanwhile, the rate of adverse analytical findings (AAF) in China remains notably higher [17].

Among the groups surveyed in our study, pharmacists showed the highest level of willingness to engage in doping prevention programs. They also reported managing consultations related to doping prevention and potential adverse drug reactions of prohibited substances. The majority of study participants identified pharmacies as the healthcare facilities where information regarding doping can be provided. 

To be a reliable source of information regarding prohibited substances and doping prevention, healthcare providers should be equipped with sufficient knowledge and skills. This education could be implemented into university curricula, such as through elective courses or as part of continuing professional development. Some universities worldwide offer a new elective course called ‘Sports Pharmacy’, which covers many topics related to doping in sports [18]. However, several limitations hinder the widespread inclusion of this course in pharmacy programs. For instance, only a limited number of institutions can provide lecturers with deep expertise in sports pharmacy or anti-doping regulations. The funding for such courses could also be challenging for some institutions. Additionally, there are insufficient opportunities for internships or practical placements in sports-focused environments, which limits students’ hands-on learning and exposure to real-world cases. To overcome these challenges, it would be a strategic move for academic institutions to collaborate with sports organizations, local anti-doping agencies, multidisciplinary departments, and WADA. Such partnerships could enhance course content, provide access to expert instructors, and facilitate practical training opportunities for pharmacy students. Collaborations with WADA-accredited laboratories would be especially beneficial in providing students with direct experience in doping control and analysis.

Several studies in the scientific literature have assessed pharmacy students’ knowledge about doping, prohibited substances, and sports pharmacy [19,20,21]. A research study conducted at the Qatar University College of Pharmacy revealed that while students recognized the importance of pharmacists in doping prevention, the majority, 60%, did not know anything about the WADA, and 85% had never heard of the International Pharmaceutical Federation’s (FIP) statements regarding the pharmacist’s role in anti-doping activities. Notably, 90% agreed to the inclusion of sports pharmacy in the curriculum, illustrating the need for formal education in the field [19]. In Norway, a study revealed that only 22.5% of pharmacy students had received training in sports pharmacy, and 91.7% expressed a desire to gain higher competence in this area [21]. A survey among Japanese pharmacy students revealed that while most were familiar with the term “doping”, a significant number had not attended lectures on the subject. Notably, 72% expressed a desire to receive such education, indicating a gap in the current curriculum and a need for enhanced educational programs to prepare future pharmacists for roles in doping prevention [20]. Findings from these studies demonstrate that despite a lack of sufficient knowledge, pharmacy students show a great interest in being involved in doping prevention and building competence within this field. Our results show that surveyed pharmacy students achieved a greater mean score (27.66 ± 4.11) compared to medical students (*p* < 0.001). This can be explained by the fact that additional subjects such as pharmacognosy, pharmaceutical chemistry, pharmaceutical analysis, and pharmaceutical care are covered in pharmacy curricula, which allow pharmacy students to acquire in-depth knowledge of medicines and DSs and ensure patient safety. As a limitation of our study, we can mention that it covers a single medical university in Bulgaria. However, the number of participating students is relatively high.

In the last decade, the unintentional doping has become a serious medical, ethical, and sports concern [22]. The numerous cases of unintentional doping gained the attention not only of the professional sports society but also of many scientists all over the world. Recently, many AAFs have been reported to be a result of unintentional doping after the intake of contaminated DSs [22]. DSs are widely used among professional athletes to enrich their diet and represent an important part of their nutritional regime. Moreover, many products containing vitamins and minerals, amino acids, ecdysterone, *Tribulus terrestris* extracts, and others are associated with the potential to enhance health, performance, and recovery. At the same time, in the last two decades, many DSs were reported to be contaminated with prohibited substances, including anabolic steroids, diuretics, sibutramine, and other pharmacologically active compounds [22]. Numerous studies have quantified the actual concentrations of these contaminants. Geyer et al. revealed that 14.8% of 634 analyzed non-hormonal supplements contained undeclared anabolic steroids, including norandrostenedione and androstenedione, with doses reaching up to 190 µg. For instance, ingesting more than 1 µg of nandrolone prohormones from such DSs is sufficient to cause an AAF under WADA testing thresholds [23]. In 2016, Krivohlavek et al. reported a concerning trend in Croatia, finding sibutramine in 20% of the 123 DSs analyzed. The highest concentration detected was 26.41 mg/g [24]. Even a single ingestion of a contaminated supplement can result in detectable concentrations in biological fluids. The elimination half-life of sibutramine is approximately 14 to 19 h. Consequently, sibutramine and its metabolites may remain detectable in urine for up to 48 h following ingestion [25].

Real-life examples further underscore this concern. LaShawn Merritt, a United States Olympic gold medalist in track and field, tested positive for DHEA after using an over-the-counter product (“ExtenZe”), which contained undisclosed anabolic steroids. Merit had received a 21 month ban, even after providing proof of the source, due to the WADA code’s strict liability principle [26]. Similarly, in 2025, U.S. boxer Isabella Winkler faced a six-month cessation sanction after testing positive for propylhexedrine, a muscle relaxant available in inhalers. Although the substance was ingested unintentionally, she was sanctioned for doping [27]. These cases highlight the fact that even non-performance-enhancing supplements, when taken in combination with commonplace drugs, can lead to inadvertent doping violations.

Currently, the regulation of DSs remains insufficiently strict, with no mandatory requirement for manufacturers to submit quality control data. In Bulgaria, DSs are regulated in alignment with EU legislation (Directive 2002/46/EC), but national enforcement remains relatively limited. In 2021, Bulgaria introduced a regulation requiring DSs to be distributed only through registered community pharmacies and food outlets, in an effort to reduce access to unregulated and potentially contaminated products. The regulation also restricts labeling practices that imply medicinal properties unless explicitly authorized. These measures were partly introduced to curb the circulation of potentially dangerous or misleading DS products, particularly those sold online without proper quality controls. However, pre-market quality testing for banned substances is still not mandatory [28,29].

In contrast, Germany employs more rigid measures, including quality control and post-marketing surveillance. In the Netherlands, the NVWA system monitors and publicly recalls DSs when necessary. In the United States, under the 1994 Dietary Supplement Health and Education Act (DSHEA), the use of supplements is not subject to pre-approval by the FDA. This creates fragmentation in consumer protection and facilitates the proliferation of contaminated products [30,31,32].

According to the Bulgarian Anti-Doping Center, DSs are not necessarily safe or free from contamination, even if their labels suggest otherwise. The center warns that DSs can pose significant doping risks due to either the presence of prohibited substances or contamination during production. They note that the use of DSs has contributed to numerous positive doping samples. For example, the Bulgarian Anti-Doping Center has provided statistics showing that 44% of positive doping samples reported by the British National Anti-Doping Agency were linked to DSs. During the Winter Olympics in Sochi, seven out of 2631 doping tests returned positive results, all of which were caused by the supplement use [33].

Athletes must receive constant education about the hidden risk of unintentional doping and how it could be prevented. Most of the athletes are not familiar with the risks associated with DS intake. It is considered that an essential key point in the strategies for the prevention of unintentional doping is the constant education of athletes [22]. According to our study, most of the healthcare professionals and students are aware of the potential risks associated with taking DSs (72%) and recognize ephedrine, a commonly found substance in weight loss DSs, as a prohibited substance included in the WADA list. These findings suggest that health professionals, particularly pharmacists, could provide essential information and support to athletes regarding the risks of DS intake. Our conclusions are consistent with previous research conducted by Smith-Morris et al. [34]. The study shows that pharmacist assessment could be beneficial for professional athletes, supporting them to be compliant with WADA standards. Findings revealed that following a comprehensive review of medications and supplements taken by athletes, nearly half were found to be using one or more prohibited substances. Pseudoephedrine was the most commonly identified substance associated with unintentional doping [34].

**Figure 9:**
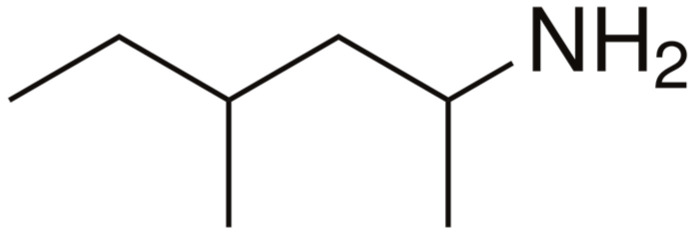
Structure of Metylhexamine.

The prevention of unintentional doping is complex and difficult. Not only do the contaminated DSs represent the risk of unintentional doping, but some good quality products of natural origin do as well by containing prohibited compounds of plant origin. In a survey among Japanese pharmacy students, over half of the respondents did not know that the most common doping violation in Japan is unintentional doping, nor were they familiar with certain past cases of doping. Furthermore, 41% were unaware that over-the-counter medicines and dietary supplements might contain prohibited substances, and 87% did not realize that names of prohibited substances might not appear on ingredient labels of dietary supplements [20]. A study conducted among 384 community pharmacists in Malaysia revealed that while there was an average level of knowledge regarding doping, a significant portion of respondents (65.9%) were unaware that unintentional doping is classified as a violation [35]. According to our study, only a small part of the medical specialists are fully prepared to address cases of unintentional doping effectively. For example, only 26.3% of the respondents are familiar with the fact that the intake of pelargonium graveolens could result in a violation of the WADA Code. In fact, recently, many local anti-doping organizations announced that the intake of DSs containing extracts from this plant could result in unintentional doping because the plant contains methylexamine (Figure 9), an indirect sympathomimetic [36]. Moreover, the compound could not be mentioned at all on the label of the DS. Some of the signals that the compound may present in the composition of DSs include a mention on the label of Geranium oil extract, Geranuim oil, Geranium extract, Geranamine, DMAA, Forthane, Forthan, Floradrene, 4-methyl-2-hexanamine, and 4-Methylhexan-2-amine, 2-amino-4-methylhe.

**Figure 10:**
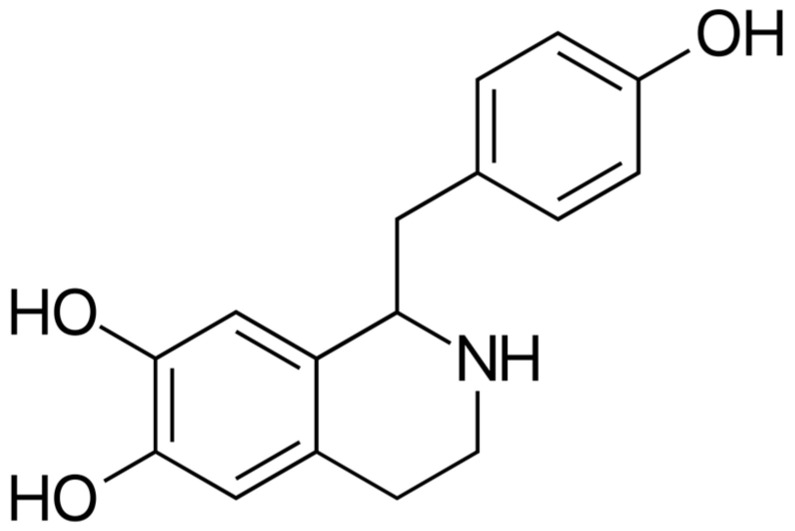
Chemical structure of 1-[(4-hydroxyphenyl) methyl]-1,2,3,4-tetrahydroisoquinoline-6,7-diol.

More than 40% of the respondents are not aware that the molecule higenamine (Figure 10) found in many plants could be regarded as a source of unintentional doping. The compound is a β_2_ agonist. It was included in WADA’s prohibited list in 2017. It could be detected in different plants and many DSs of natural origin. Some of the higenamine-containing plants include *Nelumbo nucifera*, *Tinospora crispa*, *Nandina domestica*, *Gnetum parvifolium*, *Asarum siebodii*, *Asarum heterotropoides*, *Aconitum carmichaelii*, and *Aristolochia brasiliensis* [37]. These plants are deeply involved in Eastern traditional medicine, and their intake is associated with numerous health benefits. Currently, there is a wide variety of DSs containing extracts from these plants. The supplementation regime of professional athletes is quite complex and, in general, includes many different DS intended to enrich the diet, promote health, enhance performance, and reduce the time for recovery. Without good research, DS containing higenamine could be included in the diet of professional athletes. Normally, the DS are not labeled to contain higenamine, but only the plant extracts they contain. Athletes and their teams must be aware of the issues associated with the use of plant-based DSs. In the selection of appropriate and safe DSs, pharmacists could play an important role because they have a lot of knowledge not only about chemical compounds but also about the phytochemical profiles of many plants. A crucial role for this knowledge is the discipline of pharmacognosy, which plays a special role in pharmaceutical education.

More than half of the respondents (60.1%) consider that the local community pharmacies could be involved in future doping-prevention strategies. In general, in community pharmacies, athletes can easily find support and information. Pharmacists could play an essential role in preventing doping by providing an accessible and well-informed alternative to other health professionals [38,39]. Moreover, they are recognized as the most accessible healthcare professionals.

In addition, the commitment of pharmacists in the fight against doping in sports is manifested through their active participation in educational initiatives and awareness campaigns. By raising awareness and disseminating information about the consequences and risks of doping, they contribute to an easily accessible, convenient, and more effective approach to doping prevention. One of the main advantages that pharmacists have in this field is their in-depth understanding of the safety of medicines, which allows them to tailor this knowledge to individual patient needs. This expertise enables pharmacists to provide personalized advice on the appropriate use of medicines, thus ensuring that people do not accidentally fall into the trap of doping practices. Pharmacists’ focus on the patient creates an environment of good rapport, trust, and open communication. This encourages patients to consult pharmacists on doping issues as they feel comfortable with a familiar and trusted health professional.

## 5. Conclusions

Medical specialists might play a crucial role in doping prevention strategies. The relationship between medical specialists and athletes is essential for raising awareness and information, developing life skills, and decision-making capability to prevent not only intentional but also unintentional anti-doping rule violations. However, the specialists involved in doping-prevention projects need constant and specific training about doping and doping prevention. According to this study, pharmacists are the medical specialists who are associated with the highest levels of preparedness and willingness to participate in anti-doping programs. Moreover, pharmacists are regarded as more accessible to people compared to medical doctors. The results of this study suggested that campaigns focused on doping prevention are of interest to 32.3% of the respondents, while 19.8% expressed interest but reported a lack of enough free time. Pharmacists are uniquely placed to offer valuable guidance and support to people who are concerned about doping and its consequences. According to our study, local pharmacies could also be involved successfully in doping-prevention strategies. The relationship between medical specialists and athletes could develop behaviors that foster and protect the spirit of sport.

## Figures and Tables

**Table 1:** Demographic and main characteristics of respondents.

Feature	Number	Percentage (%)
**Gender**		
Woman	371	63.4
Man	206	35.2
I prefer not to answer	8	1.4
**Age (years)**		
20–29	421	71.9
30–39	107	18.3
40–49	36	6.2
50–59	12	2.1
60+	9	1.5
**Professional status**		
Medical Doctor	122	20.9
Pharmacist	141	24.1
Medical Student	130	22.2
Pharmacy Student	192	32.8
**Sports activity**		
Current sports participation	241	41.2
Previous sports participation	261	44.6
Never practiced in sports activities	83	14.2
**Sport/sport activity category**		
Professional sports	31	5.3
Semi-professional sports	80	13.7
Amateur sports	391	66.8
No sports participation	83	14.2
**Attending a lecture about “Doping”**		
Yes	199	34.1
No	386	65.9

**Table 2:** Comparison of overall knowledge among different respondent groups regarding prohibited substances in sport and doping prevention.

Group	Number (%)	Mean Score ± SD	*p*-Value
Pharmacists	141 (24.1)	28.61 ± 4.29	0.009 *
Medical doctors	122 (20.9)	26.99 ± 5.42	
Medical students	130 (22.2)	25.30 ± 5.06	<0.001 *
Pharmacy students	192 (32.8)	27.66 ± 4.11	

* Statistically significant difference.

**Table 3:** Comparison of overall knowledge between respondents who have and have not reviewed the WADA List.

Group	Number (%)	Mean Score ± SD	*p*-Value
Respondents who have reviewed the WADA Prohibited List	111 (19.0)	30.35 ± 3.23	<0.001*
Respondents who have not reviewed the WADA Prohibited List	474 (81.0)	26.49 ± 4.84	

* Statistically significant difference.

**Table 4:** Comparison of respondents’ knowledge based on whether they practice a sport or sports activity.

Group	Number (%)	Mean Score ± SD	*p*-Value
Currently practicing sports	241 (41.2)	27.99 ± 4.65	<0.001 *
Previous sports participation	261 (44.6)	27.07 ± 4.72	
Never practiced a sports activity	83 (14.2)	25.49 ± 5.17	

* Statistically significant difference.

**Table 5:** Comparison of knowledge scores between respondents who provided consultations to athletes on doping prevention.

Indicator	Number (%)	Mean Score ± SD	*p*-Value
Consultation related to doping prevention and prohibited substances Yes No	48 (8.2) 537 (91.8)	30.06 ± 5.41 26.97 ± 4.69	<0.001 * 0.002 *
Consultation related to the side effects of prohibited substances Yes No	59 (10.1) 526 (89.9)	29.03 ± 5.30 27.02 ± 4.73	

* Statistically significant difference.

## Data Availability

The original contributions presented in this study are included in the article/supplementary material. Further inquiries can be directed to the corresponding author(s).

## Supplementary Materials

The following supporting information can be downloaded at https://www.mdpi.com/article/10.3390/pharmacy13030059/s1, File S1: Questionnaire (English); File S2: Informed Consent (English).
