# Supplementary material for: Exploring Doping Awareness: Medical Experts’ Perspectives and Their Commitment to Doping Prevention
Source: Pharmacy (Basel). 2025 Apr 24;13(3):59. doi: 10.3390/pharmacy13030059 (PMC12101216; doi:10.3390/pharmacy13030059)
Supplement: Supplementary file 1 [file pharmacy-13-00059-s001.zip › Supplementary files/Supplementary file 1.pdf]

Questionnaire

1. **Gender**
   - ☐ Male
   - ☐ Female
   - ☐ Prefer not to answer

2. **Age (years):……………..**

3. **You are:**

☐ Medical doctor
☐ Pharmacist
☐ Pharmacy student third year
☐ Pharmacy student fourth year
☐ Pharmacy student fifth year
☐ Medical student third year
☐ Medical student fourth year
☐ Medical student fifth year
☐ Medical student sixth year

4. Do you currently engage in any form of sport or physical activity?

☐ Yes, I am currently engaged in sports or physical activity.
☐ I have participated in sports or physical activity in the past, but I am not currently active.
☐ No, I have never engaged in sports or physical activity.

5. Which of the following categories best describes the level of sport or physical activity you currently engage in or have engaged in previously?

☐ Professional sport
☐ Semi-professional sport
☐ Amateur sport
☐ I do not participate in sports or physical activity

6. Have you ever attended a lecture on doping?

☐ Yes
☐ No

7. What does the term *"doping"* refer to?

☐ Consumption of specific foods
☐ A healthy diet
☐ A form of exercise
☐ The use of prohibited substances or methods to enhance athletic performance
☐ I do not know

8. **What are the two most commonly used forms of doping testing?**

- ☐ Blood sample testing
- ☐ Urine sample testing
- ☐ Hair sample testing
- ☐ Saliva sample testing

9. What is the World Anti-Doping Agency (WADA)?

- ☐ An international independent agency that monitors anti-doping activities
- ☐ An organization that promotes the use of performance-enhancing substances and methods in sport
- ☐ A committee of professional athletes
- ☐ A health organization
- ☐ I do not know

10. Have you ever seen WADA Prohibited list?

- ☐ Yes
- ☐ No

11. Do you know what blood doping is?

- ☐ Yes
- ☐ No

12. Can the use of certain dietary supplements lead to a positive doping test?

- ☐ Yes
- ☐ No
- ☐ I do not know

13. Which of the following may be considered potential indicators of doping?

- ☐ Rapid increase in muscle mass
- ☐ Enhanced endurance
- ☐ Irritability and mood swings
- ☐ Sudden and unexplained weight loss
- ☐ All of the above

14. In what ways can doping negatively impact an athlete's health?

- ☐ It can increase the risk of cardiovascular events
- ☐ It can lead to hormonal imbalances
- ☐ It can cause gynecomastia in men
- ☐ It can result in mood disturbances
- ☐ It can contribute to alopecia (hair loss)
- ☐ It can lead to organ damage

- ☐ All of the above

15. Which of the following substances are classified as doping agents? ***(Select all that apply)***

- ☐ Stimulants
- ☐ Diuretics
- ☐ Beta-2 agonists
- ☐ Caffeine
- ☐ All of the above

16. Which of the following substances are prohibited by the WADA?

- ☐ Anabolic steroids
- ☐ Alcohol
- ☐ Caffeine
- ☐ Creatinine
- ☐ Vitamins
- ☐ Ecdysterone
- ☐ All of the above
- ☐ None of the above

17. Which of the following substances is *not* classified as a doping agent?

- ☐ Vitamin D
- ☐ Corticosteroids
- ☐ Human growth hormone
- ☐ Meldonium

18. Which of the following substances is included on WADA Prohibited List due to its potential to enhance athletic performance?

- ☐ Pyridoxine hydrochloride
- ☐ Erythropoietin (EPO)
- ☐ Acetylsalicylic acid
- ☐ Ibuprofen

19. Which of the following substances is commonly misused in sport for its ability to mask the presence of other prohibited substances?

- ☐ Creatine
- ☐ Furosemide
- ☐ Vitamin D
- ☐ Lactic acid

20. Which of the following substances, frequently found in weight loss supplements, is prohibited in sport?

- ☐ Ephedrine
- ☐ Vitamin C

- ☐ Calcium
- ☐ Vitamin E

21. Which of the following substances is used to stimulate red blood cell production and is classified as a doping agent?

- ☐ Dietary supplements containing iron
- ☐ Acetylsalicylic acid
- ☐ Erythropoietin (EPO)
- ☐ Calcium supplements

22. Which of the following pain-relieving drugs is prohibited by WADA?

- ☐ Tramadol
- ☐ Acetylsalicylic acid
- ☐ Ibuprofen
- ☐ Metamizole sodium

23. Which of the following plant-derived substances are classified as prohibited in sport?

- ☐ Higenamine
- ☐ Cathinone
- ☐ Morphine
- ☐ All of the above

24. Which plant is known to be a potential source of substances banned in sport?

- ☐ *Thymus serpyllum* (thyme)
- ☐ *Pelargonium graveolens* (geranium)
- ☐ *Matricaria recutita* (chamomile)
- ☐ All of the above
- ☐ None of the above
- ☐ I do not know

25. In your opinion, why are diuretics included on WADA Prohibited List?
Please explain:
………………………………………………………………………………………

26. Are you aware of which sports prohibit the use of beta-blockers?

- ☐ Yes
- ☐ No

If your answer is "Yes," please list the sports in which beta-blockers are prohibited: ………

27. Would you be interested in participating in doping prevention campaigns?

- ☐ Yes
- ☐ I would like to, but I do not have enough free time.

- ☐ I would like to, but I do not feel I have sufficient knowledge about doping prevention or substances.
- ☐ No
- ☐ I do not know

28. Are you familiar with the sanctions imposed by WADA on athletes found guilty of doping?

- ☐ Yes, athletes may be suspended from competition for a specific period or permanently.
- ☐ Yes, athletes may receive a formal warning.
- ☐ I am not familiar with the sanctions.

29. Have you ever consulted an athlete regarding substances that enhance athletic endurance?

- ☐ Yes
- ☐ No

30. Have you ever consulted an athlete regarding the side effects associated with substances that enhance athletic endurance?

- ☐ Yes
- ☐ No

31. Are you aware of any cases of unintentional doping?

- ☐ Yes
- ☐ No

32. Do you believe it is feasible for pharmacies to serve as healthcare facilities where athletes can receive advice on the potential harms of doping?

- ☐ Yes
- ☐ No
- ☐ I do not know

33. In your opinion, where do athletes most commonly obtain banned substances? *(Please select all that apply)*

- ☐ From community pharmacies
- ☐ From online pharmacies
- ☐ From internet shops
- ☐ From fitness centers
- ☐ I do not know
- ☐ Other (please specify) __________

34. In your opinion, what are the two most important strategies for successful doping prevention?

- ☐ Organizing discussions between professional athletes, including youth, and medical professionals to share information about the side effects associated with the use of various substances commonly employed for doping.
- ☐ Implementing specialized anti-doping education programs in sports schools.
- ☐ Organizing regular discussions between professional athletes who have been sanctioned for doping and athletes from various sports clubs.
- ☐ Organizing regular talks between professional athletes who have been sanctioned for doping and students from sports schools.
- ☐ Introducing stricter penalties for athletes who use prohibited substances or methods.Strengthening regulation and quality control of dietary supplements.
- ☐ Launching educational television campaigns.

Thank you for your participation!
