# Supplementary material for: Exploring Doping Awareness: Medical Experts’ Perspectives and Their Commitment to Doping Prevention
Source: Pharmacy (Basel). 2025 Apr 24;13(3):59. doi: 10.3390/pharmacy13030059 (PMC12101216; doi:10.3390/pharmacy13030059)
Supplement: Supplementary file 1 [file pharmacy-13-00059-s001.zip › Supplementary files/Supplementary file 2.pdf]

INFORM CONSENT

Study Title: Exploring Doping Awareness: Medical Experts' Perspectives and Their Commitment to Doping prevention

Purpose of the Study: You are invited to participate in a research study assessing your knowledge of prohibited substances and methods in sports, as well as contemporary approaches to doping prevention and control. You must be at least 18 years of age and and either a pharmacy or medical student, a pharmacist, or a medical doctor.

Procedures: If you choose to participate, you will answer some questions about prohibited substances and methods in sports and your experience and willingness to participate in different antidoping activities and campaigns. The study is expected to take approximately 10 minutes.

Confidentiality: The data collected in this study are completely anonymous. No personally identifiable information will be collected and the information you choose to provide in this study cannot be connected back to you.

Voluntary Participation: Your participation in this survey is voluntary.

Contact: If you have any questions regarding this survey, please contact Vanya Rangelov Kozhuharov, PhD, Assistant Professor of Pharmaceutical Chemistry vanya.kozhuharov@mu-plovdiv.bg

Consent: I have read and understand the above consent form. By clicking the "Next" button to enter the survey, I indicate my willingness to voluntarily take part in this study.
